# Supplementary material for: Dynamics of SARS-CoV-2 Antibody Responses up to 9 Months Post-Vaccination in Individuals with Previous SARS-CoV-2 Infection Receiving Inactivated Vaccines
Source: Viruses. 2023 Apr 4;15(4):917. doi: 10.3390/v15040917 (PMC10145073; doi:10.3390/v15040917)
Supplement: Supplementary file 1 [file viruses-15-00917-s001.zip › viruses-2252277-supplementary.pdf]

Supplementary Table S1. SARS-CoV-2-specific antibody levels in SARS-CoV-2 Recovered individuals with different disease severity.

	Mild and Moderate (n = 43)	Severe (n = 15)	p values*
**WT. NAbs**			
T0	17.8	22.60	0.6286
T1	50.2	40.80	0.6577
T2	33.2	29.30	0.6730
T3	27.7	17.95	0.1589
**WT. NP IgG**			
T0	19.5	16.1	0.4230
T1	75.4	150.2	0.1000
T2	48.5	72.1	0.8387
T3	61.0	48.0	0.2383
**WT. S1 IgG**			
T0	138.4	138.9	0.8730
T1	322.7	311.2	0.9241
T2	236.4	232.1	0.9232
T3	208.7	157.2	0.1876
**Omic. NAbs**			
T0	6.6	8.3	0.9553
T1	27.5	31.3	0.7580
T2	23.6	25.0	0.9786
T3	15.1	4.4	0.0251
**Omic. S1 IgG**			
T0	42.5	59.9	0.6600
T1	154.0	137.3	0.7978
T2	92.2	87.6	0.7559
T3	89.9	63.0	0.1743

NAbs, neutralization antibodies. WT, wild-type strain. Omic, Omicron. NP, nucleoside protein.
All values are expressed as median.
* p values calculated using a two-sided Mann–Whitney U test.

Supplementary Table S2. SARS-CoV-2-specific antibody levels in SARS-CoV-2 Recovered individuals according to age.

	Age (≤ 60 years) (n = 40)	Age (＞ 60 years) (n = 18)	p values*
**WT. NAbs**			
T0	17.9	23.9	0.2691
T1	46.4	43.4	0.7580
T2	32.3	37.5	0.2346
T3	27.9	18.0	0.0662
**WT. NP IgG**			
T0	17.3	32.1	0.0146
T1	75.3	152.6	0.1013
T2	46.6	91.2	0.4838
T3	55.4	48.0	0.4547
**WT. S1 IgG**			
T0	136.5	164.5	0.2627
T1	319.5	325.8	＞0.9999

T2	228.5	235.7	0.3261
T3	214.2	172.5	0.1633
**Omic. NAbs**			
T0	5.8	14.9	0.0651
T1	25.4	36.2	0.3790
T2	13.6	30.6	0.1736
T3	21.5	4.9	0.1042
**Omic. S1 IgG**			
T0	34.1	61.2	0.3521
T1	158.6	121.4	0.5141
T2	90.8	113.6	0.5092
T3	90.8	67.3	0.1777

NAbs, neutralization antibodies. WT, wild-type strain. Omic, Omicron. NP, nucleoside protein. All values are expressed as median.

* *p* values calculated using a two-sided Mann–Whitney U test.

Supplementary Table S3. SARS-CoV-2-specific antibody levels in SARS-CoV-2 Recovered individuals according to gender.

	Male (n = 32)	Female (n = 26)	p values*
**WT. NAbs**			
T0	17.7	21.7	0.4578
T1	43.5	46.3	0.3398
T2	31.6	37.7	0.3090
T3	26.6	25.8	0.4820
**WT. NP IgG**			
T0	18.0	25.3	0.2434
T1	92.5	78.8	0.6827
T2	51.9	72.7	0.9634
T3	64.6	39.4	0.4007
**WT. S1 IgG**			
T0	126.0	174.0	0.1476
T1	311.2	340.3	0.2630
T2	223.2	243.9	0.1015
T3	176.5	194.9	0.3096
**Omic. NAbs**			
T0	7.9	6.0	0.9609
T1	26.8	36.2	0.9836
T2	20.4	41.2	0.4022
T3	13.2	14.7	0.9123
**Omic. S1 IgG**			
T0	33.6	63.6	0.1476
T1	126.7	154.0	0.4918
T2	81.5	115.4	0.2981
T3	89.9	75.6	0.6852

NAbs, neutralization antibodies. WT, wild-type strain. Omic, Omicron. NP, nucleoside protein. All values are expressed as median.

* *p* values calculated using a two-sided Mann–Whitney U test.

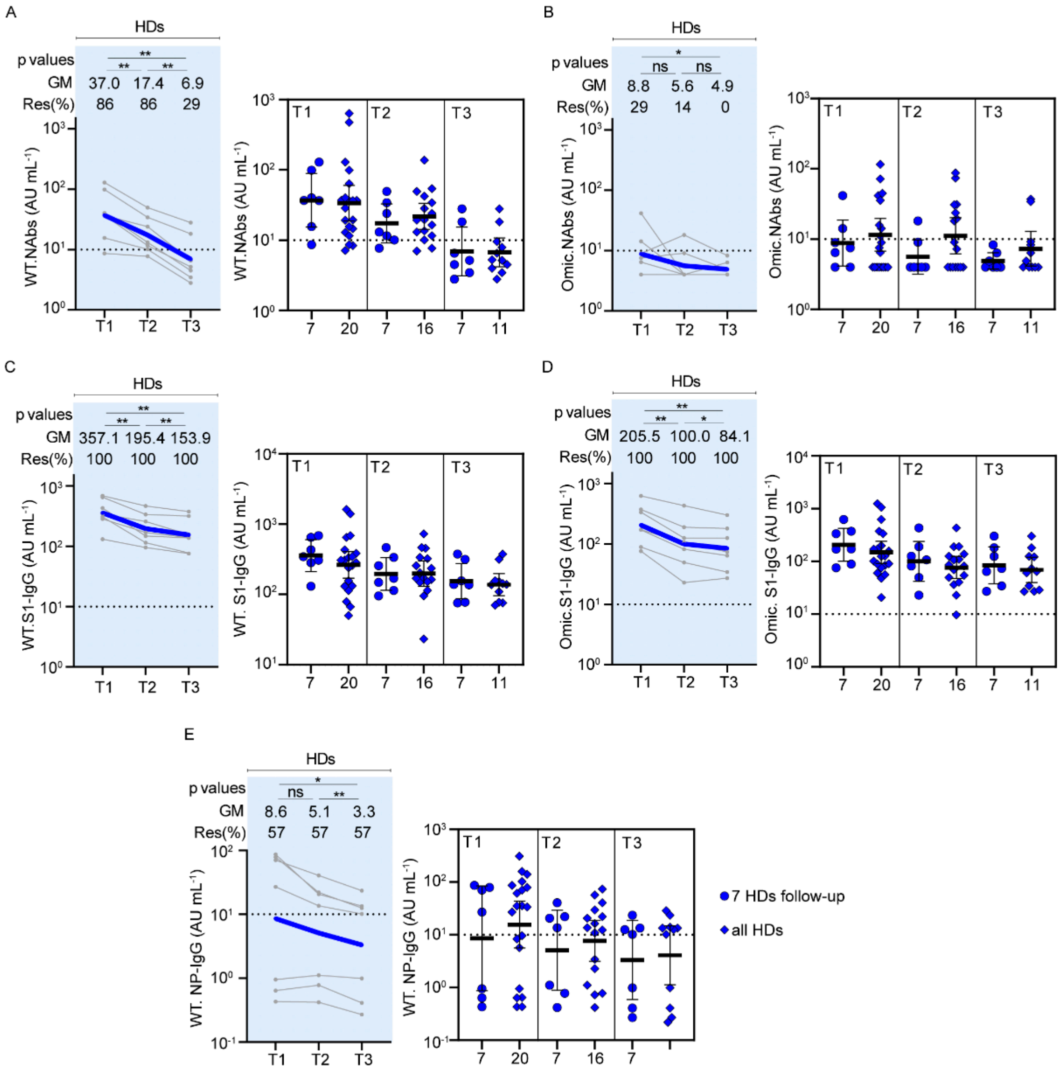

Supplementary Figure S1. Dynamics of virus-specific antibodies in 7 HDs with longitudinal follow-up. The left parts display kinetics in NAbs against wild-type strain (**A**), NAbs against Omicron (**B**), Spike S1 domain-specific antibodies against wild-type strain (**C**), Spike S1 domain-specific antibodies against Omicron (**D**), and nucleoside protein-specific IgG antibodies against wild-type strain (**E**) in 7 HDs at 3, 6, and 9 months after the 3rd vaccination. Individuals are shown as gray symbols with connecting lines. Geometric means are shown in thick blue lines. The right panel displays the comparison of antibodies between 7 HDs and all HDs over the three time points. Seven HDs with longitudinal follow-up are show in blue circles and all HDs are show in blue diamonds. Dotted lines indicate the cut-off value. Statistics were calculated using Wilcoxon's signed-rank test for paired groups and Mann–Whitney U test for unpaired groups: * $p < 0.05$; ** $p < 0.01$; *** $p < 0.001$; **** $p < 0.0001$. GM, geometric mean; Res, responders; NAbs, neutralizing antibodies; WT, wild-type strain; Omic, Omicron.

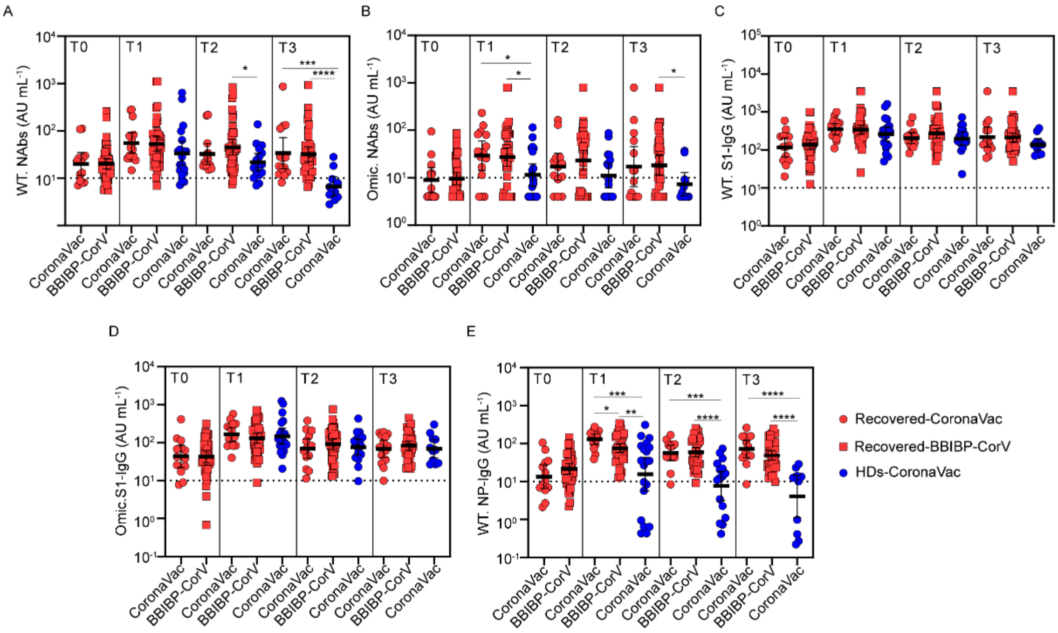

Supplementary Figure S2. SARS-CoV-2-specific antibody levels in individuals with previous SARS-CoV-2 infection with different vaccination. The scatter diagrams show the comparison of NAbs against wild-type strain (A), NAbs against Omicron (B), Spike S1 domain-specific antibodies against wild-type strain (C), Spike S1 domain-specific antibodies against Omicron (D), and nucleoside protein-specific IgG antibodies against wild-type strain (E) in SARS-CoV-2 Recovered individuals receiving CoronaVac (shown in red circle), SARS-CoV-2 Recovered individuals receiving BBIBP-CorV (shown in red square), and HDs receiving CoronaVac (shown in blue circle). Dotted lines indicate the cut-off value. Statistics were calculated using Wilcoxon's signed-rank test for paired groups and Mann–Whitney U test for unpaired groups: * $p < 0.05$; ** $p < 0.01$; *** $p < 0.001$; **** $p < 0.0001$. NAbs, neutralizing antibodies; WT, wild-type strain; Omic, Omicron.

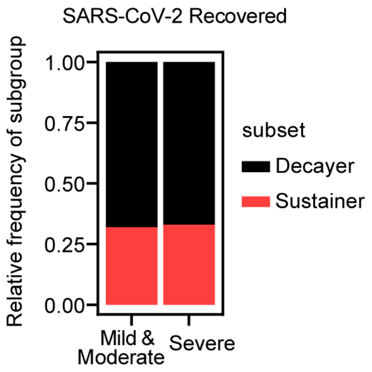

Supplementary Figure S3. Frequency of Sustainer and Decayer subsets in different disease severity. Sustainers are displayed in red and Decayers in black color.

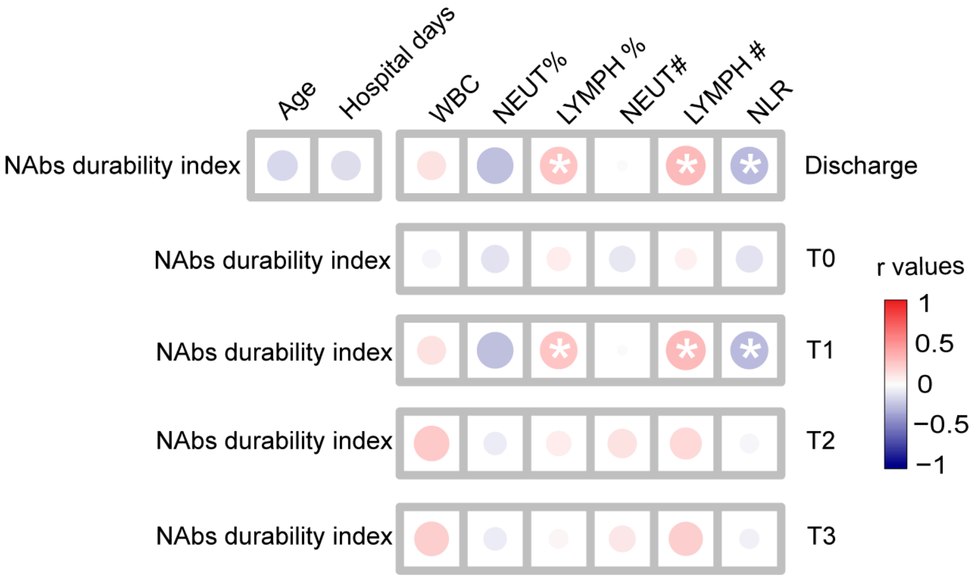

Supplementary Figure S4. Correlation analysis between NAbs durability index and clinical variables in individuals with previous SARS-CoV-2 infection. The correlation matrixes of antibody levels are calculated using nonparametric Spearman rank correlation in individuals with previous SARS-CoV-2 infection. Positive correlations are displayed in red and negative correlations in blue color. The size and color of each dot in the triangular matrix show the strength of correlation between the variables: * $p < 0.05$; ** $p < 0.01$; *** $p < 0.001$; **** $p < 0.0001$. NAbs, neutralizing antibodies. WBC, white blood cell. NEUT %, neutrophil %. LYMPH %, lymphocyte %. NEUT #, neutrophil counts. LYMPH #, lymphocyte counts. NLR, neutrophil-to-lymphocyte ratio.
